# Allograft Reconstruction of the Extensor Mechanism after Resection of Soft Tissue Sarcoma

**DOI:** 10.1155/2018/6275861

**Published:** 2018-05-22

**Authors:** Daniel A. Müller, Giovanni Beltrami, Guido Scoccianti, Pierluigi Cuomo, Francesca Totti, Rodolfo Capanna

**Affiliations:** ^1^Department of Orthopedic Surgery, Balgrist University Hospital, Forchstrasse 340, 8008 Zürich, Switzerland; ^2^Department of Orthopedic Oncology, Azienda Ospedaliero-Universitaria Careggi, Largo Brambilla 3, 50134 Firenze, Italy; ^3^Department of Orthopedic Surgery, Azienda Ospedaliero-Universitaria Pisana, Via Paradisa 2, Cisanello, 56125 Pisa, Italy

## Abstract

**Introduction:**

Soft tissue tumors around the knee joint still pose problems for the excision and subsequent reconstruction.

**Methods:**

In the 6 included patients the soft tissue sarcoma has its base on the anterior surface of the extensor mechanism and expands towards the skin. The entire extensor apparatus (quadriceps tendon, patella, and patellar tendon) was resected and replaced by a fresh-frozen allograft.

**Results:**

The mean follow-up was 6.7 years (range: 2–12.4 years). In two patients a local recurrence occurred, resulting in a 5-year local recurrence-free rate of 66.7% (95% CI: 19.5%–90.4%). Distant metastases were found in 4 patients resulting in a 5-year metastasis-free rate of 33.3% (95% CI: 4.6%–67.5%). Two patients underwent at least one revision surgery, including one patient in whom the allograft had to be removed. According to the ISOLS function score 24.7 points (range: 19–28 points) were achieved at the last follow-up. The mean active flexion of the knee joint was 82.5° (range: 25–120°) and a mean extension lag of 10° (range: 0–30°) was observed.

**Conclusions:**

The replacement of the extensor mechanism by an allograft is a reasonable option, allowing wide margins and restoration of active extension in most patients.

**Trial Registration:**

The presented study is listed on the ISRCTN registry with trial number ISRCTN63060594.

## 1. Introduction

Soft tissue sarcomas represent a heterogeneous group of lesions that are often subtle in presentation and have a wide variation in extent of malignant behavior. Because of the rarity and vast histological varieties, they frequently pose diagnostic problems. The knee joint and the adjacent structures are quite uncommon locations for soft tissue sarcoma [1]. The most common entities are liposarcoma (25%) and synovial sarcoma (25%), followed by fibrosarcoma (9%) and undifferentiated pleomorphic sarcoma (8%) [1].

Over the recent decades, limb-sparing surgery has become an accepted method of treatment for most patients with soft tissue sarcomas. Advances in surgical techniques, prosthetic design, and bone allograft banking have opened the avenue for reconstruction procedures after wide excision of bone and soft tissues. This allows the surgeon to achieve adequate margins while salvaging the limb and its function. Yet, soft tissue tumors around the knee joint still pose problems for the excision and subsequent reconstruction. Extension of the tumor in the joint or in the extensor mechanism is a challenging situation to achieve adequate margins and a good functional limb. There is no current consensus as to the best method of reconstruction of the extensor mechanism of the knee [2]. Few reconstructive options are available to avoid knee arthrodesis including tendon transfers or local grafts [3], synthetic materials [4], and allograft tissues [5]. Regardless of the used reconstruction technique, the principal technical difficulty remains to be restoration of the extensor function and the provision of soft tissue coverage. In the current literature, no data is available considering specifically the demanding situation of soft tissue sarcomas involving the extensor mechanism of the knee. This study aims to highlight the surgical difficulties as well as the oncological and functional results after a resection of the extensor apparatus due to a soft tissue sarcoma.

## 2. Materials and Methods

### 2.1. Patients

**Figure 1:**
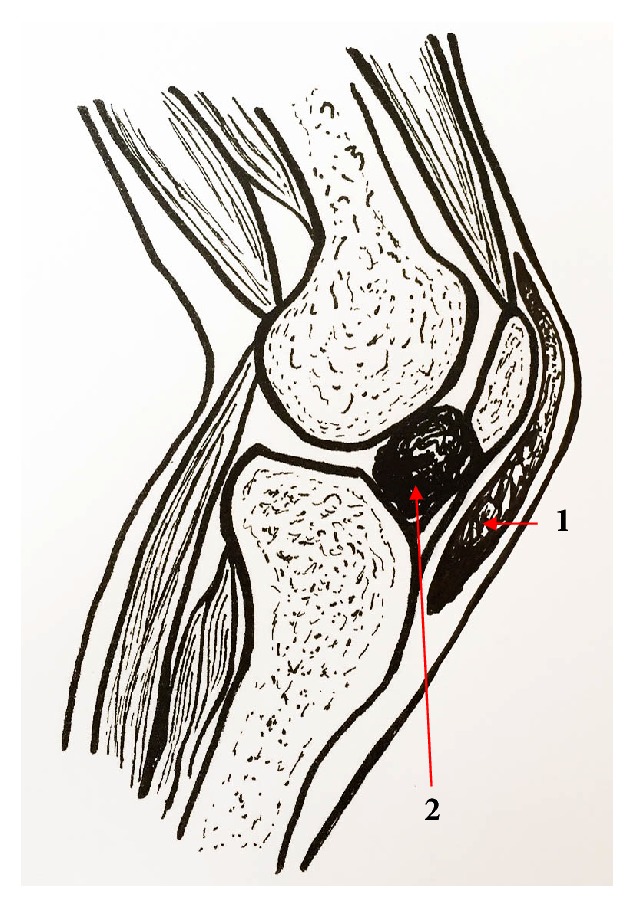
*Inclusion criteria of the patients according to the tumor localization.* Schematic illustration of a knee joint (lateral view). The patients were divided into 2 groups according the tumor localization. Patients with a superficially spreading lesion and no joint involvement were included in this study (indicated as number 1). If the tumor site is deep and involves the joint space (indicated as number 2) an extra-articular resection of the knee was performed.

Between 2002 and 2012 17 patients were treated for a soft tissue sarcoma involving the knee extensor apparatus in one institution. We observed mainly two different extension and growth patterns of the tumor (see Figure 1): 11 patients showed an intra-articular expansion of the tumor mass with contamination of the joint cavity, whereas in 6 patients the soft tissue sarcoma has its base on the anterior surface of the extensor mechanism and expands towards the skin. The 11 patients with involvement of the joint space needed an extra-articular knee resection and an allograft prosthetic composite reconstruction as formerly described [6] and were therefore excluded from this study.

All patients (*n* = 6), who have undergone a surgical resection of a soft tissue sarcoma affecting the quadriceps tendon, the patella, and/or the patellar tendon without apparent joint involvement were included in this study. The tumors were adherent and fixed to the underlying extensor mechanism.

These cases were often misdiagnosed as a chronic inflammation process of the prepatellar bursa prior to the referral to our institution. Therefore, all the included patients underwent an inadequate and intralesionally previous surgery before our treatment. The presence of either microscopic (R1) or macroscopic (R2) residual tumor was classified as inadequate resection. The histological diagnosis was based on the primary excision of the tumor. All the external histology samples were ordered and reviewed by the institutional pathology. The detailed characteristics of the included patients are indicated in Table 1.

### 2.2. Surgical Technique

It must be assumed that tumor cells were implanted in the surrounding tissue after the first inadequate surgery outside. During the reexcision the whole area which is theoretically contaminated should be removed. In all the included cases the previous surgeries were small with short incisions. The presented surgical technique was only performed, if the whole previous surgical area could be removed completely. If this was not possible, an extra-articular resection or an above-knee amputation was considered.

Only in the patient who underwent an arthroscopy the area of the possible iatrogenic spread of tumor cells was difficult to define. As the tumor was outside the joint capsule and not touched by the arthroscopy portals, we considered a limb salvage procedure feasible. However, it is important to bear in mind that a compartmental resection was not achieved in this case with the presented technique. The only remaining alternative surgical option would be an above-knee amputation, which the patient refused categorically.

**Figure 2:**
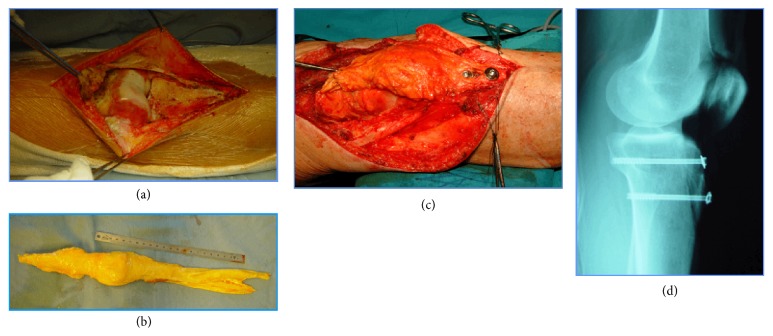
*Surgical technique.* Clinical case (patient number 4) as example for the surgical technique used in the described study population. (a) Complete resection of lesion and extensor apparatus; (b) preparation of the osteotendinous allograft; (c) fixation of the allograft with screws distally and direct end-to-end suture proximally; (d) postoperative radiography.

The crucial criterion for the surgical indication in the presented patients was the absence of tumor involvement of the joint cavity. All the presented patients had an MRI before the initial surgery in the referral hospital. The MRI before and after the initial inadequate surgery were both available and evaluated in our institution. If no effusion was found in the clinical examination and all available MRI revealed no tumor mass deep to the extensor mechanism, we resigned an extra-articular knee resection. Instead, the whole extensor apparatus was resected in its entire length including distal part of quadriceps tendon, patella, and patellar ligament through a midline anterior approach (Figure 2). The quadriceps tendon was spared as much as the tumor expansion allowed. In all patients, a size-matched fresh-frozen nonirradiated allograft harvested from donors younger than 40 years was used to restore the extensor mechanism. The allograft consists of the quadriceps tendon, the patella, the patellar tendon, and the tibia tuberosity. First the allograft bone block of the tuberosity was fixed to the host tibia by screws. Then the allograft quadriceps tendon was sutured to the remaining host tendon and to the medial and lateral edges of the quadriceps muscle in full extension of the knee joint. This important tendon to tendon suture was done by modified Mason Allen stitches [7] with nonabsorbable fibers.

In 5 out of 6 patients, a free or rotational flap was necessary for sufficient soft tissue coverage: a rotational medial gastrocnemius flap was performed in one and a free fasciocutaneous flap (anterolateral thigh flap from the contralateral limb) in 4 patients.

### 2.3. Surgical Aftercare and Follow-Up

The affected knee was maintained in a plaster splint for 5 weeks. After removal of the splint, patients began active and passive mobilization assisted by a physiotherapist. For the first 5 weeks, an ambulation with 2 crutches and non-weight bearing was allowed. The weight bearing was increased stepwise until full weight bearing was usually achieved 3 months after the surgery. The patients were followed with serial clinical and radiographic examinations of the limb, combined with CT imaging of the chest. For the first 3 months, clinical and radiographic follow-ups were obtained monthly. Afterwards the intervals were extended to 3 months in the first 2 years, 4 months in the 3rd year, 6 months in the 4th, 5th, and 6th years, and finally once a year until the 10th year after the surgery.

### 2.4. Oncological Treatment

The indication and the sequence of adjuvant therapies such as chemotherapy or irradiation were discussed in a multidisciplinary sarcoma board. Compared to other soft tissue sarcomas, synovial sarcomas are relatively chemosensitive [8]. For this reason, both patients diagnosed with synovial sarcoma received a systemic treatment with doxorubicin and ifosfamide. Patient number 3 had a high grade pleomorphic sarcoma with a high probability of developing metastatic disease. The interdisciplinary board decided to apply a preoperative “prophylactic” chemotherapy and radiation therapy. In patient numbers 1 and 2 the surgical margins were either marginal or tumor-free but “close.” These two patients received a postoperative radiation therapy to improve local control.

### 2.5. Measurements and Statistical Analysis

As indicators for the knee function the active range of motion, active flexion, and extension lag were measured and the International Society of Limb Salvage (ISOLS) score was calculated [4] at the last follow-up.

Survival curves were generated using the Kaplan-Meier method. Statistical analyses were done by the help of the software Stata version 12.1 (Statacorp, College Station TX, USA). *P* values < 0.05 were considered significant.

## 3. Results and Discussion

### 3.1. Results

The mean follow-up was 6.7 years (range: 2–12.4 years). An overview considering the complications of the treatment and oncological outcome is highlighted in Table 2.

#### 3.1.1. Complications

In 2 patients, at least one revision surgery was necessary including 1 patient in whom the allograft had to be removed: Patient number 1 fell on the operated knee 12 years after the surgery and suffered consecutively from an extension deficit. A rupture of the quadriceps tendon was identified at the musculotendinous transition zone. The ruptured tendon was replaced by a new allograft of the extensor apparatus and reinforced by an artificial ligament. The second case who needed a surgical revision (patient number 6) showed a partial necrosis of the primary flap during the first two weeks postoperatively. A secondary coverage was achieved by skin transplantation harvested from the inguinal region.

In one case (patient number 2) a traumatic fracture of the allograft happened 6 years after the surgery, affecting the tibia tuberosity. As the fracture showed no displacement and the integrity of the extensor mechanism was not disturbed, conservative treatment achieved complete healing of the bone.

#### 3.1.2. Functional Results

The different functional outcomes for every patient are indicated in Table 3. On average 24.7 points (range: 19–28 points) were achieved using the ISOLS score. The mean active flexion of the knee joint was 82.5° (range: 25–120°). Patients were suffering from a mean extension lag of 10° (0–30°). Due to this lag, the range of motion averaged with 72.5° (range: 25–100°) lower than the flexion alone. No obvious correlation between the preoperative (all the patients had a ROM of at least 90° and more before the surgery in our institution) and the postoperative function was found. The poor ROM of patient number 6 was due to the difficult soft tissue situation. The primary flap was necrotic and a revision surgery was performed. A complete closure of the surgical field was achieved, but the flexion was restricted because of scar tissue and adhesions.

#### 3.1.3. Oncological Results

At the last follow-up one patient was disease-free, 2 patients were alive with disease and 2 patients died because of the disease. In one patient no evidence of disease was found after treating a local recurrence. The disease specific 5-year survival of the patients was 62.5% (95% CI: 21.3–89.3%).

The histological examination of the resected tumor revealed 5 wide surgical margins and one case with marginal margins. In all patients, residual tumor cells were found in the reexcision samples. Especially in the deep surface against the extensor mechanism the margins were all inadequate, probably because the previous surgeons were afraid to injure the patellar ligament.

**Figure 3:**
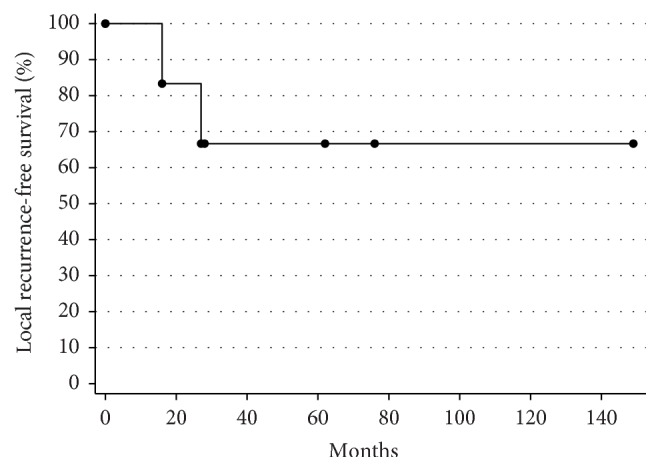
*Local recurrence-free survival curve.* Kaplan-Meier curve for local recurrence-free survival.

The patient with marginal margins had a local recurrence after 16 months. Furthermore, one local recurrence occurred after an adequate surgery with wide margins. As shown in Figure 3 the 5-year local recurrence-free rate is 66.7% (95% CI: 19.5%–90.4%). Distant metastases were found in 4 patients resulting in a 5-year metastasis-free rate of 33.3% (95% CI: 4.6%–67.5%).

The effect of the applied radiotherapy for local control and the chemotherapy for systemic disease was not measurable as the numbers are too small and the histologic types of the sarcomas are too heterogeneous. The radiation therapy was applied as planned with the favored dose in all 3 patients concerned. As the tumors were all superficial, skin reactions were common. But no complication due to the radiation therapy was observed.

### 3.2. Discussion

A soft tissue sarcoma of the extensor mechanism poses a particularly demanding surgical situation. The oncologic surgeon does handle the difficulties not only to achieve adequate margins in a surgical field of sparse soft tissue coverage, but also to reconstruct the extensor mechanism providing good functional results.

The aim of the study was to evaluate the (1) complications, (2) functional outcome, and (3) oncological results in patients suffering from a soft tissue sarcoma of the knee extensor mechanism with absence of joint invasion.

#### 3.2.1. Limitations

Our presented case series has some limitations. First, as with other series dealing with reconstruction of the knee extensor mechanism, the number of included patients is small, although relatively large compared to the available studies in the literature. Second, our series of tumors was heterogeneous for type, stage, and adjuvant treatment. These variables certainly affect rates of survival but not whether adequate margins or restored function was achieved. Third, different procedures for soft tissue coverage were performed, according to the case-specific situation.

#### 3.2.2. Reconstruction Technique

The use of an allograft extensor mechanism proved to be a valid strategy for the reconstruction. The presented technique is mainly for primary tumors without any previous surgery. These are the preferred conditions for an oncological correct resection providing the best local control. Experience in using an allograft extensor mechanism in the tumor setting is lacking completely in literature. Although in a nononcological setting (major revision in total knee arthroplasties), previous reports confirm the safety and functionality of the reconstructive procedure [2, 9–12]. Our functional results regarding active range of motion, active flexion, and extension lag are consistent with these series [2, 9, 10, 12]. There is only one existing case study which can be compared to our results. Imanishi et al. described 4 cases [5] in whom the patellar tendon was resected due to a soft tissue sarcoma. The patellar tendon was reconstructed with a bone-tendon-bone allograft and fixed by wires and screws to the residual patella and tibia tuberosity. This technique presupposes the preservation of the quadriceps tendon together with some residual patella. In all our patients this would have led to inadequate excision of the tumor. The functional results were excellent in the series of Imanishi et al. [5] as all patients achieved virtually full range of motion and 28–30 points in the MSTS score. The difference to our results probably lies in the extent of the resection. All of our patients had to undergo a complete resection of the previous surgical area. In addition to the defect of the extensor mechanism, large soft tissue defects resulted. The need of extensive resections certainly has a negative influence on the functional outcome.

Different alternative reconstructions were previously described in the oncologic setting. As substitute for the patellar tendon a fascia lata graft [3, 13], autologous achilles tendon [14], or hamstring tendon grafts [15, 16], as well as a latissimus dorsi flap [17], were proposed. In contrast to all these case reports Muramatsu et al. [18] described a complete resection of the extensor apparatus including quadriceps tendon, patella, and patellar tendon. After treating the resected specimen with liquid nitrogen intraoperatively, the extensor mechanism was reimplanted as recycled autograft. We have some reservations concerning this recycled autograft technique, as in our opinion the oncologic safety is questionable.

#### 3.2.3. Complication Rate

In our presented reconstruction technique, the short- and long-term morbidity rates were acceptable for tumors, which widely involved the knee. No deep infection was observed. Possible explanation is the use of an aggressive perioperative antibiotic prophylaxis. Two of the three complications were attributed to the allograft: one allograft fracture and one rupture of the quadriceps tendon. The fracture was treated conservatively, meaning that a total of one allograft (16.7%) needed to be surgically revised. This is slightly higher than previous reported case in revision surgeries of failed extensor mechanism due to total knee arthroplasties [19]. Fracture and rupture of the allograft both occurred late in clinical course and had clear traumatic reason. Assuming the healing was complete after this time, the complications are hardly attributable to the reconstruction technique. Nevertheless, Imanishi et al. [5] observed a fracture of the allograft, too. So, the structural weakness of the allograft substitute seems to be the major reason for failure.

#### 3.2.4. Oncologic Outcome

The residual tumor and the whole area of the previous surgery were removed in one piece allowing a reliable histological work-up. In all the included patients an above-knee amputation was a valid option. In general, the safety of tumor removal must always be weighed against the remaining limb function. In all the presented patients we decided against an amputation after a comprehensive discussion.

However, it is not clear whether amputation would have improved the oncological result. The metastatic rate cannot be influenced by local measures and depends mainly on size and histologic grading of the tumor [20]. Regarding the local recurrence current studies showed that the outcome is worse if an unplanned excision in a nononcological setting was done prior to the referral in a tertiary sarcoma center, independent of the anatomical location [21–23].

The oncological outcome is difficult to compare with other series in the literature as no specific results for the extensor mechanism are available and the prognostic among soft tissue sarcomas varies widely according to histologic type, grade size, and location [24–27]. Local recurrence rates after radiation and resection have been reported as high as 20% [28]; however most recent series report number at 10% or less [24, 25, 29]. In overall, our local recurrence rate seems to be much higher compared to other localizations. All patients have undergone an inadequate previous surgery before the referral to our tertiary care center. Because of the superficial location of the lesions and the proximity to the prepatellar bursa, they were all misdiagnosed as benign tumors or chronic bursitis. An unplanned excision of soft tissue sarcomas or an inadequate biopsy is associated with a poor local control [21–23]. At least in our series, unplanned resections of soft tissue sarcomas before a correct diagnosis seem to pose a serious problem around the knee joint. One patient had even undergone an arthroscopy before the sarcoma diagnosis. Although the arthroscopic procedure was independent of the tumor excision, there is still the risk of wide spreading of tumor cells through the used liquid. At the time of the reexcision in our institution no evidence for joint involvement was found but the decision for a joint-preserving resection remains debatable in this situation. Unfortunately, a local recurrence occurred indeed, and the patient died from the disease due to systemic metastasis.

A recent published case study reported similar oncologic problems: 3 out 4 patients included in their series had an unplanned excision of a sarcoma affecting the patellar tendon [5]. But, the present case series is inhomogeneous with respect to former surgery and the potential contamination status before the index operation in our institution. This may additionally cause the bias of high local recurrence rates.

## 4. Conclusions

In summary, the treatment of soft tissue sarcoma involving the extensor apparatus of the knee is associated with a high rate of previous inadequate surgeries and high local recurrence and metastatic rates. The replacement of the extensor mechanism by an allograft is a reasonable option, allowing wide margins and restoration of active extension in most patients.

## Figures and Tables

**Table 1:** Characteristics of included patients.

Patient number	Diagnosis	Localization	Previous surgery	Margins at previous surgery	Chemotherapy	Radiotherapy	Flap
1	Myxofibrosarcoma	Patella	Inadequate excision	R1	None	Postoperative	Anterolateral thigh, free flap
2	Myxofibrosarcoma (recurrent)	Patellar tendon	Inadequate excision	R1	None	Postoperative	Anterolateral thigh, free flap
3	Pleomorphic sarcoma (recurrent)	Patellar tendon	2 inadequate excisions	R2	Postoperative	Preoperative	Medial gastrocnemius
4	Synovial sarcoma (recurrent)	Patellar tendon	Arthroscopy; 2 inadequate excisions	R2	Pre- and postoperative	None	None
5	Synovial sarcoma	Patella	Inadequate excision	R2	Preoperative	None	Anterolateral thigh, free flap
6	Pleomorphic sarcoma	Patella	Inadequate excision	R1	None	None	Anterolateral thigh, free flap

**Table 2:** Oncological results and complications.

Patient number	Follow-up (months)	Oncological result	Local recurrence (months)	Metastasis (months)	Complications
1	149	CDF	None	None	Rupture of quadriceps tendon
2	145	NED	27	None	Fracture of tibia tuberosity
3	62	DOD	None	15	None
4	22	DOD	16	17	None
5	76	AWD	None	6	None
6	28	AWD	None	6	Partial necrosis of flap

CDF: continuously disease-free; DOD: dead of disease; AWD: alive with disease; NED: no evidence of disease.

**Table 3:** Functional results.

Patient number	Follow-up (months)	ISOLS score (points)	Active ROM (degree °)	Active Flexion (degree °)	Extension Lag (degree °)
1	149	28	60	90	30
2	145	19	90	100	10
3	62	28	100	120	20
4	22	23	60	60	0
5	76	24	100	100	0
6	28	26	25	25	0

ROM: range of motion.
